# Involving patients and caregivers to develop items for a new patient‐reported experience measure for older adults attending the emergency department. Findings from a nominal group technique study

**DOI:** 10.1111/hex.13811

**Published:** 2023-06-30

**Authors:** Blair Graham, Jason E. Smith, Ffion Barham, Jos M. Latour

**Affiliations:** ^1^ School of Nursing and Midwifery, Faculty of Health University of Plymouth Plymouth UK; ^2^ Department of Emergency Medicine University Hospitals Plymouth NHS Trust Plymouth UK; ^3^ School of Nursing, Midwifery and Paramedicine, Faculty of Health Sciences Curtin University Perth Western Australia Australia

**Keywords:** consensus, emergency department, nominal groups, patient experience

## Abstract

**Context:**

Patient experience is an important component of high‐quality care and is linked to improved clinical outcomes across a range of different conditions. Patient‐reported experience measures (PREMs) are psychometrically validated instruments designed to identify where strengths and vulnerabilities in care exist. Currently, there is no validated instrument available to measure patient experience among people aged over 65 years attending the emergency department (ED).

**Objective:**

This paper aims to describe the process of generating, refining and prioritising candidate items for inclusion in a new PREM measuring older adults' experiences in ED (PREM‐ED 65).

**Design:**

One hundred and thirty‐six draft items were generated via a systematic review, interviews with patients and focus groups with ED staff exploring older adults' experiences in the ED. A 1‐day multiple stakeholder workshop was then convened to refine and prioritise these items. The workshop entailed a modified nominal groups technique exercise comprised of three discrete parts—(i) item familiarisation and comprehension assessment, (ii) initial voting and (iii) final adjudication.

**Setting and Participants:**

Twenty‐nine participants attended the stakeholder workshop, conducted in a nonhealthcare setting (Buckfast Abbey). The average age of participants was 65.6 years. Self‐reported prior experiences of emergency care among the participants included attending the ED as a patient (*n* = 16, 55.2%); accompanying person (*n* = 11, 37.9%) and/or as a healthcare provider (*n* = 7, 24.1%).

**Results:**

Participants were allocated time to familiarise themselves with the draft items, suggest any improvements to the item structure or content, and suggest new items. Two additional items were proposed by participants, yielding a total of 138 items for prioritisation. Initial prioritisation deemed most items ‘critically important’ (priority 7–9 out of 9, *n* = 104, 75.4%). Of these, 70 items demonstrated suitable inter‐rater agreement (mean average deviation from the median < 1.04) and were recommended for automatic inclusion. Participants then undertook final adjudication to include or exclude the remaining items, using forced choice voting. A further 29 items were included. Thirty‐nine items did not meet the criteria for inclusion.

**Conclusions:**

This study has generated a list of 99 prioritised candidate items for inclusion in the draft PREM‐ED 65 instrument. These items highlight areas of patient experience that are particularly important to older adults accessing emergency care. This may be of direct interest to those looking to improve the patient experience for older adults in the ED. For the final stage of development, psychometric validation amongst a real‐world population of ED patients is now planned.

**Patient and Public Contribution:**

Initial item generation was informed using qualitative research, including interviews with patients in the ED. The opinions of patients and members of the public were integral to achieving outcomes from the prioritisation meeting. The lay chair of the Royal College of Emergency Medicine participated in the meeting and reviewed the results of this study.

## 1 INTRODUCTION

Patient experience is an important component of high‐quality, patient‐centred care and is associated with improved outcomes for a range of acute conditions including pneumonia, acute coronary syndrome and asthma.^1^, ^2^, ^3^ Older adults currently account for about a quarter of emergency department (ED) attendances and this proportion is likely to increase further given the ageing global population.^4^, ^5^ Older adults may have a range of additional care requirements and psychosocial needs when accessing emergency care, compared to younger adults.^6^, ^7^ Capturing older adults' experiences of care may identify where vulnerabilities and subsequent opportunities for improvement in the provision of emergency care exist.

Patient‐reported experience measures (PREMs) are validated, self‐reported questionnaires that are directly reported by patients and aim to provide standardised evaluation of individual experiences of care. PREMs differ from patient‐reported outcome measures (PROMs), which measure patients' views of their health status, and satisfaction surveys, which measure to what extent care meets patients' subjective expectations.^8^, ^9^ Hodson and Roberts^10^ suggest that patient satisfaction measures often exhibit a ceiling effect, whereby responses are predominantly positive. Hence, satisfaction surveys may be less likely to identify negative determinants of experience compared to PREMs. This is important, as negative determinants of experience may represent particularly useful areas for performing quality improvement. As such, the use of PREMs to capture patient experiences of emergency care is suggested within the International Federation of Emergency Medicine framework for quality and safety in Emergency Medicine.^11^ However, a systematic review of existing PREMs in emergency care determined that there was significant variation in the quality of existing instruments, including uncertain validity, reliability and responsiveness.^12^ These findings are reflected in a further systematic review of 88 PREMs which reported inconsistent adherence to established criteria for the selection of health instruments.^13^, ^14^ Recently, PREMs have been developed to capture older people's experience of hospital and community care, although no instrument specific to the ED yet exists.^15^, ^16^

The PREM for patients attending the ED, aged over 65 (PREM‐ED 65) aims to address the current gap, by developing and validating a PREM for use in older adults accessing emergency care. The first stage of PREM‐ED 65 development aimed to generate a comprehensive understanding of determinants of older adults' experiences of receiving ED care. Initially, a systematic review of qualitative studies was conducted leading to the formulation of a conceptual framework for patient experience in the ED.^17^ This framework highlighted the importance of meeting patients' communication, emotional, care, physical/environmental and waiting needs. Confirmation of conceptual validity and expansion of the framework was then achieved by undertaking semi‐structured interviews with older adults during an emergency care episode, and focus groups with staff responsible for the provision of emergency care to older adults across three EDs.^18^, ^19^

This study aims to describe the process of generating and prioritising a list of suggested items for PREM‐ED 65 by involving multiple stakeholders including patient and public representatives, healthcare professionals and advocates for older adults.

## 2 MATERIALS AND METHODS

### 2.1 Item generation

An initial list of candidate items was developed by two researchers (B. G. and J. M. L.) following methodological triangulation of findings from prior studies conducted by the research team. These consisted of a qualitative metasynthesis of 22 studies of patient experience in the ED^17^; interviews conducted with 24 patients aged over 65 attending the ED^18^; and interprofessional focus groups with 37 ED staff.^19^ Methodological triangulation describes the use of multiple data sources to study a phenomenon, and is useful to confirm findings, enrich data and increase overall validity.^20^ Therefore, similar findings that occurred across more than one of the studies were identified as particularly relevant as a focus for future measurement of older adults' experiences of ED care. Item generation focused on these recurrent areas. To enrich understanding, excerpts of relevant findings were highlighted, extracted and grouped together. Each group of excerpts was then summarised by the two researchers and translated into a single suggested item for inclusion in PREM‐ED 65. To ensure the conceptual underpinnings of the study were respected, the research team discussed the meaning of each item and categorised each item according to one of the five analytical themes: communication, emotional, waiting, care needs, physical and environmental needs, or team attitudes and behaviours.

Following identification, the wording of each of the draft items was subjected to a readability assessment, accomplished by calculating a Flesch Reading Ease (FRE) score. The FRE provides a simple formula for assessing semantic difficulty and is commonly used to interpret the readability of health information.^21^ The score signifies how easy a statement is to read on a scale of 0 (*most difficult* [postgraduate reading level]) to 100 (*least difficult* [9‐year‐old reading level]). Typically, a score of 70 is assumed to be accessible to the average adult.^22^ In practical terms, this represents the reading age of an average 12‐year‐old. Therefore, candidate items with a score of less than 70 at the initial assessment were modified by simplifying the vocabulary, syllable count and structure of the statement. Readability was considered satisfactory when a postadjustment score of greater than about 70 was attained.

### 2.2 Prioritisation of items

**Figure 1:**
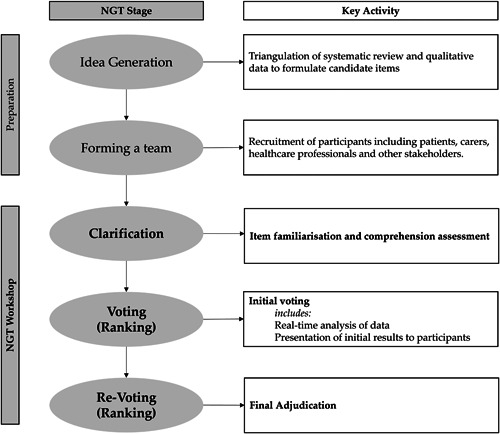
Flow diagram demonstrating the NGT process to develop items for PREM‐ED 65. ED, emergency department; NGT, nominal groups technique; PREM, patient‐reported experience measure.

A 1‐day workshop was held with multiple stakeholders (*n* = 29) to prioritise the list of candidate items. The day was structured using an adaptation of the nominal groups technique (NGT). The NGT provides a recognised method of gaining group consensus using a combination of discussion and voting. A particular advantage of NGT over other consensus methods is that it can provide a prompt result.^23^, ^24^ The workshop programme consisted of (i) item familiarisation and comprehension assessment, (ii) initial voting and (iii) final adjudication (Figure 1).

A range of approaches was used to recruit a convenience sample of patients, carers, health professionals and relevant third‐party stakeholders. This included e‐mail advertisements to members of patient groups affiliated to local hospitals, clinical research departments and the ambulance service. Information posters were also displayed in three participating EDs. In addition, the lead researcher (B. G.) promoted the workshop to members of the public at a research engagement event during September 2019, directly approaching stakeholders including relevant charities advocating for older adults (Age UK; Healthwatch) and the lay committee of the Royal College of Emergency Medicine. Upon receipt of an initial expression of interest, potential participants were emailed a formal electronic invitation consisting of a participant information sheet, written consent form and registration form. Participants were issued with joining instructions on receipt of their registration form.

The workshop was held in the conference facilities of a nonhealthcare setting (Buckfast Abbey), in December 2019. No incentive was offered but refreshments including lunch were provided, and participants' travel expenses were reimbursed.

The workshop programme was designed to minimise both participant burden and the potential for respondent fatigue during prioritisation exercises. It was recognised that some participants would be living with frailty or disability and provisions for ease of access were ensured during planning. The pace of sessions was monitored by five facilitators distributed throughout the room, and extended breaks were provided.

The study received prospective ethical approval from the University of Plymouth Faculty of Health Research Integrity & Ethics Committee (1920/1173).

#### 2.2.1 Item familiarisation and comprehension assessment

For the first workshop exercise, participants were asked to provide a comprehensibility assessment of items. For each item, participants were asked to determine whether the item was (i) ‘easy to read’ (Yes/No) and (ii) ‘easy to understand’ (Yes/No). Participants were invited to suggest new items if any gaps were identified.

#### 2.2.2 Initial voting

The second workshop exercise was initial prioritisation. During this voting exercise, participants were presented with each item and asked to individually vote on the perceived importance for inclusion in PREM‐ED 65. This was accomplished using a nine‐point interval scale; priorities 1–3 were labelled ‘less important’, priorities 4–6 as ‘Important, but not critical’ and 7–9 were ‘Critically Important’.

The median priority and measure of inter‐rater agreement (absolute deviation from the median [ADM]) was calculated for each item.^23^, ^25^ The mean ADM (MADM) across all items was then calculated, and individual items with an ADM greater than 50% of the mean value were deemed as having insufficient inter‐rater agreement. This was used to determine whether the item was eligible for inclusion, exclusion or final adjudication in a second round of voting (Table 1). Data collection and analysis for initial voting was accomplished in real‐time by members of the research team (F.B. and B.G.) using a preformulated instrument developed in Microsoft Excel.

**Table 1:** Criteria for initial prioritisation.

Priority to include item in PREM‐ED 65^(median score/item)^	Inter‐rater agreement^(MADM)^	Outcome
7–9 (Critical)	Sufficient	Include item
	Insufficient	Final adjudication
3–6 (Important, but not critical)	Any	
1–3 (Not important)	Insufficient	
	Sufficient	Exclude item

*Note*: Insufficient Inter‐rater agreement threshold = MADM > 50%.

Abbreviations: MADM, mean absolute deviation from the median; PREM, patient‐reported experience measure.

#### 2.2.3 Final adjudication

The third workshop exercise was the final adjudication. This consisted of dichotomous voting for items which did not meet inclusion or exclusion criteria during the first round. During this exercise, participants were presented with the item and requested to vote to either ‘include’ or ‘exclude’ the item. To facilitate inclusion of only those items for which there was clear positive consensus, a majority threshold of at least 75% was prospectively agreed to determine the criteria for inclusion. This threshold is comparable with other studies.^26^, ^27^

#### 2.2.4 Participant evaluation

Participants were invited to complete an optional 10‐item anonymised paper‐based survey at the end of the workshop. This aimed to evaluate overall satisfaction with the NGT process, the ability to meaningfully participate and invite suggestions for future improvements.

## 3 RESULTS

### 3.1 Initial item generation

One hundred and thirty‐six suggested items were derived following triangulation of findings from the metasynthesis, interviews with patients and focus groups with ED staff. Compared to the original conceptual framework, candidate items most frequently aligned to the themes of communication needs (33 items), care needs (33 items) and emotional needs (27 items). A smaller number of items concerned waiting needs (18 items), physical and environmental needs (15 items) and team attitudes and values (10 items).

Each of the initial 136 suggested items was tested against the FRE score. The median FRE score for the 136 items preadjustment was 67.3 (range: 11–100), equating to a reading age of about 15 years. Items with a score of less than 70 (*n* = 68) were individually adjusted with the intention of increasing readability. Adjusted items were then reviewed by the researchers to ensure meaning and construct validity was maintained. Following the adjustment of items, the median FRE score of the participants increased to 80.3 (range: 66–86). The initial list of candidate items is available in Electronic Supporting Information Material S1.

### 3.2 Workshop participants

Twenty‐nine participants attended the consensus workshop (Table 2). The median age of professional participants was 55 years (range: 32–58 years) and lay participants was 73 years (range: 63–82 years). Eighteen participants (62.1%) were female. The majority were from a managerial or professional background (72.4%, *n* = 21). Participants were surveyed on any previous engagement with emergency care. Twenty‐seven participants (93%) had experience of emergency care either as a patient (*n* = 16, 55.2%) and/or as an accompanying person (*n* = 11, 37.9%). A further seven (24.1%) participants reported experiences as a health professional, and eight (27.6%) in another professional role, for example, as a third‐sector representative from a patient advocacy organisation. Other experiences (*n* = 14, 48.2%) included voluntary positions in the ED, with affiliated charities and research ‘patient and public involvement’ group members. Additionally, 11 (37.9%) participants reported currently receiving care for at least one long‐term health condition. Participant characteristics are summarised in Table 2.

**Table 2:** **Participant characteristics**.

Characteristic	*n* (%)
Gender	
Male	11 (37.9)
Female	18 (62.1)
Age (years)	
<35	1 (3.4)
36–55	4 (13.8)
56–65	4 (13.8)
66–75	15 (51.7)
76–85	3 (10.3)
Not disclosed	2 (6.9)
Median age	71 years
Professionals	55 years
Lay participants	73 years
Occupation	
Not specified	4 (13.8)
Unskilled or semi‐skilled	0 (0)
Skilled or technical	1 (3.4)
Professional or managerial	21 (72.4)
Voluntary/honorary role	3 (10.3)
Personal experience of emergency care^a^	
Yes	29 (100.0)
As patient	16 (55.2)
As accompanying person	11 (37.9)
As health professional	7 (24.1)
As third sector worker	8 (27.6)
Other	14 (48.3)
Personal experience of long‐term condition	
Yes	11 (37.9)
No	14 (48.3)
Not disclosed	4 (13.8)

^a^ Sum of responses does not equal 100% as participants were asked to report all experiences of emergency care.

### 3.3 Item familiarisation and comprehension assessment

To reduce the burden on participants, the 136 items were divided between four groups (34 items/group). Each group was facilitated by either a member of the study team or a volunteer who was a final‐year medical student. All facilitators received prior training in the study protocol and NGT method. Group members were encouraged to assess allocated items for comprehension using a ‘think aloud’ technique, led by a group facilitator.^28^ All items were retained and were assessed as being easy to comprehend. Two additional items were added and agreed between participants, both following a large group discussion relating to the perceived importance of recognising disabilities in the ED (Quotations 1 and 2).

My disability did not get in the way of my care.

Staff recognised my hidden disability.

Quotations 1 and 2: Additional items suggested by participants.

As a result, a final list of 138 items was generated.

### 3.4 Initial voting

The final list of 138 items underwent initial prioritisation. Each workshop participant rated the priority of each of the items using the predetermined nine‐point scale.

The median priority assigned to items was 8 out of 9 (range: 1–9, interquartile range = 6). Most items were considered ‘critically important’ (priority 7–9, *n* = 104, 75.9%). Only four items (3.1%) were considered ‘less important’ (priority 1–3). The remaining items were ‘important but not critical’ (priority 4–6, *n* = 29, 21.1%).

Items meeting the threshold for the satisfactory inter‐rater agreement were eligible for automatic inclusion or exclusion in the first round. This was calculated as <50% of the overall mean average deviation from the median (MADM, <1.04).

Real‐time data analysis of first‐round prioritisation data yielded 70 (50.7%) items meeting criteria for automatic inclusion in PREM‐ED 65 (priority 7–9 and MADM < 1.04). By way of example, the highest ranking 10 items are presented in Table 3. All remaining items (*n* = 68, 49.2%) required further voting; this included the four items identified as less important, as inter‐rater agreement was insufficient to justify automatic exclusion.

**Table 3:** Top 10 ranking items included via initial prioritisation (presented in rank order based on median priority and then inter‐rater agreement (MADM).

Item	Median priority	MADM
Staff who were learning were always supervised.	9	0.11
The pain relief medicine worked well.	9	0.19
I could trust the A&E staff.	9	0.3
Pain relief medicine was brought to me quickly.	9	0.3
Staff were thorough and paid attention to the finer details.	9	0.33
Someone asked me about my views on being revived should my heart stop.	9	0.44
The A&E team were respectful and polite.	9	0.46
My disability did not get in the way of my care.	9	0.46
I felt like staff had reached the right diagnosis.	9	0.48
Staff undertook checks to make sure my skin was not at risk of damage.	9	0.48

Abbreviations: A&E, accident & emergency (ED); MADM, mean average deviation from the median.

### 3.5 Item final adjudication

The 68 remaining items were subjected to final adjudication. Of these, 39 (57.3%) items received insufficient favourable votes, resulting in their suggested exclusion from the PREM‐ED 65. The lowest ranked 10 items are presented in Table 4. Notably, all four of the items originally prioritised as ‘less important’ were excluded during this round (average proportion of ‘favourable’ votes for these items, 32.4%).

**Table 4:** Bottom 10 ranking items, excluded via final adjudication.

Item	(Round 1 findings)		Favourable votes (%)
	Median priority	MADM	
Members of the team such as house‐keeping staff and cleaners were helpful.	7	1.56	30
Members of the team appeared well rested.	6	1.59	30
Staff had a good sense of humour.	4	1.68	30
I was given a say in whether I was admitted.	3	1.93	30
I felt in control of my own situation.	6	1.07	22
Waiting in A&E is not too frustrating.	5	2.11	22
I was aware of how the urgency of my problem compared to other patients also in A&E.	4	2.11	15
Staff recognised if I had a special event such as a birthday.	2	1.82	11
The department was not too busy or hectic.	5	2.15	0
I could chat or speak with other patients.	1	1.11	0

Abbreviations: A&E, accident & emergency (ED); MADM, mean average deviation from the median.

### 3.6 Final prioritised list of candidate items for inclusion in PREM‐ED 65

An additional 29 items were prioritised for inclusion because of final adjudication. Hence, a total of 99 out of 138 items remained eligible for inclusion in the instrument, representing 71.7% of the original items.

The finalised full prioritised list of included and excluded items are presented in Electronic Supporting Information Material S2.

### 3.7 Participant evaluation

A total of 27 out of the original 29 participants (93.1%) returned completed evaluation surveys. Overall satisfaction with the NGT workshop was high among all groups, extending to the quality of the information provided during the day (100% ‘Good’/‘Very Good’), perceived relevance of the day to prioritising experience in the ED (100% ‘Agree’/‘Strongly Agree’), and ability to engage/‘have an adequate say’ during the day (100% ‘Agree’/‘Strongly Agree’).

## 4 DISCUSSION

This paper describes the process of generating and prioritising a list of candidate items for the PREM‐ED 65. There is currently no accepted gold standard for generating or prioritising items for inclusion in either PROMs or PREMs, despite this being an essential step to ensuring face validity, content validity and representativeness of items to the target population. Approaches include reviews of existing similar instruments, generation of expert consensus, interviews, use of focus groups and patient/public involvement strategies such as the utilisation of special interest groups.^29^, ^30^, ^31^, ^32^ Previous studies have confirmed the successful use of NGT both among populations of older people and multiple stakeholders.^33^, ^34^, ^35^, ^36^, ^37^

PREM‐ED 65 represents the first instrument to attempt to measure older peoples' experiences of ED care. We defined our intended PREM user group based on numeric age, as this provides the single most convenient and accessible inclusion criteria to facilitate routine usage of the PREM amongst older adults in ED settings. An age exceeding 65 years is commonly used to identify older people in the UK setting.^38^ A multiple methods approach has been employed for the generation and prioritisation of items. This aims to produce an item set that captures all potentially relevant determinants of experience for the intended population. Methodological triangulation of the literature, and primary qualitative data from both patient interviews and professional caregivers, succeeded in generating a comprehensive list of suggested items that is well aligned to the original ‘needs‐based’ conceptual framework of ED patient experience. Presentation of the items to multiple stakeholders confirmed comprehensibility and indicated that the original list was likely to be representative of older peoples' experiences in the ED. The emergence of two additional items, through group discussions, ensures that PREM‐ED 65 will measure recognition of disabilities amongst older adults accessing emergency care. This may be important, particularly as the prevalence of disability increases with age. For example, self‐reported disability among the UK population in 2022 was 9% in childhood, rising to 59% in adults aged over 80 years.^39^ Specific to emergency care, Tanderup et al.^40^ included the presence of disability as a discrete geriatric condition when evaluating characteristics of older adults attending an ED in Denmark. In this study, the presence of one or more geriatric conditions was associated with poorer health outcomes following ED attendance. Furthermore, improving transitions from ED care to community settings may prevent functional decline and increased disability that occurs in older adults following ED attendance.^41^, ^42^

Our experience is that conducting NGT amongst a population of older adults is an achievable and rewarding means to effectively prioritise items for inclusion within a PREM. Using this approach it was possible to assess and prioritise all items within a single day. To this end, NGT may be more efficient than other consensus‐building methods, most notably the Delphi method, where ongoing participant engagement is required during multiple asynchronous rounds of voting, often spanning months in duration. This requires high levels of participant engagement throughout the process, to avoid attrition.^43^ Furthermore, NGT may yield the highest levels of accomplishment and satisfaction compared to either the Delphi method or unstructured groups.^44^ This is reflected in the high satisfaction reported amongst participants in this study, as reported through postevent feedback.

For the NGT, the first round prioritisation revealed that most candidate items were deemed of ‘critical’ importance. Therefore, the method was effective in identifying very high‐priority items for inclusion in the instrument—that is, those assigned 7–9 out of 9 and meeting the predetermined criteria for inter‐rater agreement. The highest‐ranking items related to themes including supervision of trainees, effectiveness of pain management, trustworthiness and communication skills of caregivers. Specific to older adults, participants agreed that assessment of tissue viability (‘staff undertook checks to make sure my skin wasn't at risk of damage’) was of critical importance. The latter is reflected in recent literature, highlighting that prolonged ED length‐of‐stay is independently associated with the development of hospital‐acquired pressure sores. In the current international context, where ED crowding and prolonged length‐of‐stay is the norm, adequate tissue viability assessment and pressure sore prevention during the ED stay is essential.^45^ Additionally, the importance of many of the other themes are prominently recognised in the literature. For example, stakeholders within this study were almost unanimous in emphasising the importance of clinical supervision for trainees in ensuring an optimal experience. Indeed, supervision of trainees in the ED has been recognised as essential to both ensuring patient safety, and facilitating clinicians' professional development.^46^ In relation to pain management, older people may be more susceptible to receiving inadequate pain relief in the ED, compared to younger patients.^47^

Although the first round of voting was very effective in highlighting items for inclusion, it was not possible to exclude any item using this initial round, and it was, therefore, necessary to proceed to a round of dichotomous voting. Through the application of forced choice, it was possible to identify 38 items for exclusion. Examples of themes related to the lowest ranking items related to social communication (e.g., ‘I could chat or speak with other patients’), perceptions of the ED environment and patient empowerment.

The exclusion of unnecessary, unhelpful or otherwise redundant candidate items represents an important stage in the development of user‐friendly health surveys. It is generally recognised that overly lengthy or cumbersome health surveys negatively affect participant engagement, potentially contributing to nonresponse bias, incomplete responses and satisficing to ‘reduce the cognitive burden of choosing’.^48^, ^49^ Each of these factors may adversely affect the validity of results, potentially compromising instrument credibility.^50^ Furthermore, shortened questionnaires have been shown to effectively measure experiences of care.^51^ The NGT has provided an initial means of reducing items for PREM‐ED 65.

To validate the psychometric properties of PREM‐ED 65, a quantitative study will be conducted with a population of ED patients. This study will aim to confirm how each item performs in a real‐world setting by assessing participant engagement, floor/ceiling effects and differential validity of the items. Any items with low engagement or problematic validity will be removed to reduce the length of the questionnaire. The remaining items will undergo exploratory factor analysis to confirm structural validity. Additionally, the study will assess the internal consistency of measurement scales and test–retest reliability. The goal is to make PREM‐ED 65 suitable for assessing the experiences of a wide range of older adults in the ED.

### 4.1 Limitations

The generation of candidate items from the primary literature and qualitative data is based on subjective interpretation. Participant engagement in the workshop activities was adequate throughout, and the aims achieved.

We utilised multiple recruitment channels to include opinions from various stakeholders. We were mindful of promoting inclusivity among older adults in attendance by carefully selecting the venue and workshop programme. However, we acknowledge the limitations of convenience sampling. Notably, all participants in our study were White British and mostly from higher socioeconomic backgrounds (professional/managerial occupations). This apparent lack of diversity is reflective of the demography of the study locality, but nonetheless may affect the generalisability of results to ethnic minority groups, as well as individuals with limited literacy, and those from lower socioeconomic backgrounds. As an inclusive patient‐public workshop, we did not measure participants' level of frailty or use this as an inclusion criterion for the study; however, we recognised the possibility that severely frail people may be underrepresented in our sample. We aimed to mitigate this potential bias by including participants who were carers or professional advocates for people living with severe frailty, such as the manager of a dementia care centre, an older peoples' falls service lead, nursing and allied health professionals. As it remains important for PREM‐ED 65 to capture the experiences of the diverse population of older adults attending the ED, recruitment of a representative cross‐section of older adults attending the ED will be prioritised during psychometric validation.

In our study, initial voting did not eliminate items. We suggest that actively encouraging nuanced discussion between participants, during the clarification stage of the NGT, may help enable differentiation of items earlier in the process. The lower priority assigned to some aspects of patient experience during final adjudication is incongruent with the importance assigned within the literature or by interview or focus group participants. Notably, workshop participants deprioritised items related to social interactions, shared decision making and physical comfort within the ED waiting room. This may be related to the sampling issues already discussed, but also potentially the phenomenon of rosy retrospection, which describes the cognitive tendency to both anticipate events and view the past more positively than was encountered.^52^ As such, it is possible that some aspects of experience—such as the comfort of waiting room chairs, or the friendliness of staff—assume a much greater importance whilst ‘living’ an ED experience, as opposed to abstracting an experience during a workshop conducted in a nonhealthcare setting.

General concerns related to group‐based idea generation include individual dominance, ‘groupthink’, where a desire for group harmony impedes the generation of new ideas, or ‘peer pressure’, where fear of criticism may have a similar effect. The nominal group technique effectively aims to limit these phenomena, by incorporating a combination of independent ideas generation, group discussion and individual voting. Specifically, nominal groups discourage a ‘single train of thought’ as might occur in unstructured group discussions.^53^ Crucially, all participants in this study reported that they felt able to have an adequate say during the course of the workshop.

## 5 CONCLUSIONS

This paper describes a straightforward process for generating and prioritising candidate items as part of the development of an outcome measure instrument. The techniques described may be applicable to the development of other PREMs, PROMs and health surveys. The nominal group technique is both an effective and efficient method for identifying and prioritising critically important items for an instrument. However, forced choice adjudication may be necessary as a means of confirming items that are potentially redundant or unnecessary.

Findings from this study highlight areas of patient experience that are likely to be particularly important to older adults when attending the ED. In particular, the themes contained within the highest priority candidate items may be of direct interest to clinicians and policymakers concerned with improving the experiences of older adults accessing emergency care. In general, ongoing research is required to confirm the most reliable means to generate and prioritise items for inclusion in patient‐reported measures. This is necessary to ensure optimum face validity, content validity and reliability of all future instruments. As for PREM‐ED 65, the final stage of development will consist of psychometric testing amongst a population of older adults attending the ED.

## AUTHOR CONTRIBUTIONS

Blair Graham conceived and led the design of the study. Jos Latour and Jason E. Smith assisted with the design of the study. Blair Graham and Ffion Barham undertook data collection during the workshop. Blair Graham led the analysis and interpretation of data, and the development of the article. All authors contributed to the interpretation of the data and finalised the article.

## CONFLICT OF INTEREST STATEMENT

The authors declare no conflict of interest.

## ETHICS STATEMENT

This study received institutional approval from the University of Plymouth (19/20 1173). Written informed consent was obtained from all study participants.

## Supporting information

Supporting information.

Supporting information.

## Data Availability

The data that support the findings of this study are available from the corresponding author upon reasonable request.
