# Supplementary material for: Involving patients and caregivers to develop items for a new patient‐reported experience measure for older adults attending the emergency department. Findings from a nominal group technique study
Source: Health Expect. 2023 Jun 30;26(5):2040–9. doi: 10.1111/hex.13811 (PMC10485325; doi:10.1111/hex.13811)
Supplement: Supplementary file 1 — Supporting information. [file HEX-26--s001.docx]

| Table S1: Table of candidate items by theme (post- readability assessment) |  |
| --- | --- |
| 1. Candidate items related to *communication needs* |  |
| 1. Staff spoke to me as a person |  |
| 1. Staff were kind |  |
| 1. Staff introduced themselves by name |  |
| 1. The staff spent enough time speaking with me |  |
| 1. Staff made the right amount of eye contact. |  |
| 1. Staff informed me if something did not go to plan |  |
| 1. Staff ensured that I had heard what they had said |  |
| 1. Staff met my cultural and language needs. |  |
| 1. Staff involved my relatives or carers as much as I wanted. |  |
| 1. I understood what was being said |  |
| 1. Staff checked that I understood what they had said. |  |
| 1. Leaflets or pictures were used to help me understand. |  |
| 1. Staff explained everything in enough detail. |  |
| 1. Staff explained the possible outcomes of tests |  |
| 1. Staff let me know what the diagnosis might be. |  |
| 1. Staff understood my point of view. |  |
| 1. I received regular updates. |  |
| 1. Information helped to reassure me. |  |
| 1. I was informed whether I was likely to be admitted or discharged home |  |
| 1. Staff helped me feel sure that I would be able to cope at home. |  |
| 1. Staff told me when I should be well enough to get back to the things I normally do in life. |  |
| 1. Staff told me when I should be well enough to get back to my normal level of mobility. |  |
| 1. I did not have to repeat myself to many different staff. |  |
| 1. I could ask the questions I wanted |  |
| 1. Answers to my questions were clear |  |
| 1. Staff gave me clear discharge instructions |  |
| 1. Staff let me know when to return if things get worse. |  |
| 1. Staff gave me a leaflet or information sheet to take home. |  |
| 1. Staff gave me a discharge letter to give to my GP |  |
| 1. Staff had a good sense of humour. |  |
| 1. Staff checked how I would like to be addressed |  |
| 1. Staff were friendly and cheerful. |  |
| 1. I could chat with other patients if I wanted. |  |
| 1. Candidate items related to *emotional needs* |  |
| 1. I did not feel like I was treated differently because of age |  |
| 1. Staff let me know how sick I was |  |
| 1. I felt that staff understood my worries and concerns. |  |
| 1. I felt like I was treated with respect |  |
| 1. I felt like I was safe during my A&E stay |  |
| 1. I had confidence in the care I received |  |
| 1. I was able to make my own decisions about my care |  |
| 1. Staff recognised if I had a special event such as a birthday |  |
| 1. I was encouraged to walk around if I wanted to |  |
| 1. I was given a say in whether I was admitted or discharged |  |
| 1. Staff informed me why I was being admitted |  |
| 1. I felt able to make my own choices about my care |  |
| 1. I was helped to feel in control of my own situation. |  |
| 1. Staff made sure I got exactly what I needed in A&E. |  |
| 1. I did not feel lonely during my time in A&E |  |
| 1. I did not feel vulnerable during my A&E stay |  |
| 1. My relatives or carers did not get in the way of my care. |  |
| 1. Staff asked about my ideas, my concerns and my expectations of care. |  |
| 1. I was given a call bell or other means of summoning help |  |
| 1. Being in A&E was stressful for my relatives or carers. |  |
| 1. Staff reassured me. |  |
| 1. Staff were thorough and paid attention to the finer details |  |
| 1. Staff cared for my emotional needs. |  |
| 1. I did not leave A&E feeling frightened or scared about my condition. |  |
| 1. I could trust the A&E staff |  |
| 1. I felt like staff had reached the right diagnosis |  |
| 1. I felt safe to be discharged |  |
| 1. Candidate items related to *care needs* |  |
| 1. I was asked how much pain I was in | |
| 1. My pain levels were checked more than once | |
| 1. Pain relief medicine was brought to me quickly | |
| 1. The pain relief medicine worked well for me. | |
| 1. I was told why I needed medicine and about side effects. | |
| 1. I was given some choice about the type of medicine to take, such as tablets or a drip | |
| 1. Staff made effort to relieve my shortness of breath | |
| 1. Staff made effort to relieve my other symptoms | |
| 1. Staff undertook checks to make sure my skin wasn’t at risk of damage | |
| 1. Staff explained what is likely to be causing my symptoms | |
| 1. Staff took notice of my long term conditions | |
| 1. Staff told me whether I could take my usual medications whilst in A&E | |
| 1. Someone asked me for my views on life support treatment should my condition get worse | |
| 1. Someone asked me about my views on being revived should my heart stop | |
| 1. My dignity was always protected | |
| 1. I was helped to the toilet | |
| 1. Staff were quick to respond when I asked for help with the toilet. | |
| 1. Staff let me know why I needed a procedure. | |
| 1. Staff explained what they were doing to me. | |
| 1. Staff asked for my consent before they did anything | |
| 1. Staff explained what I was required to do during a procedure | |
| 1. Staff explained the risks of tests and procedures | |
| 1. Staff let me know in advance when a procedure was likely to be painful or cause me discomfort | |
| 1. The pain I felt during procedures was about the same, or was less, than I was initially told | |
| 1. Staff who were learning were always supervised. | |
| 1. I did not have to wait too long for tests or procedures | |
| 1. I was monitored and observed for the right amount of time | |
| 1. I could see usually see a clock if I wanted to check the time | |
| 1. There were enough windows and natural light in A&E | |
| 1. Staff were quick to respond to my problems | |
| 1. Staff were attentive to my needs | |
| 1. Staff were competent | |
| 1. Staff informed me when they were unsure | |
| 1. Candidate items related to *waiting needs* | |
| 1. I had adequate privacy during my A&E stay | |
| 1. I felt safe and secure whilst waiting | |
| 1. I did not feel intimidated by the other patients in A&E | |
| 1. There were enough seats in the waiting room to go around | |
| 1. I was not required to wait in the corridor for a long period | |
| 1. I was looked after whilst waiting | |
| 1. I was aware of how busy the rest of the A&E department was, whilst waiting | |
| 1. Waiting in A&E was not too frustrating | |
| 1. I was given an estimate of how long I would have to wait when I was seen at triage. | |
| 1. There were enough staff on duty | |
| 1. The department was not too busy or hectic | |
| 1. The waiting room chairs were comfortable | |
| 1. Reception desks were easy to find | |
| 1. There were activities for me to do whilst waiting so that I did not become bored. | |
| 1. Staff kept me informed about waiting times | |
| 1. I was aware of how the urgency of my problem compared to other patients also in A&E. | |
| 1. I felt like I was a priority | |
| 1. The waiting room was calm, relaxed and pleasant | |
| 1. Candidate items related to *physical and environmental needs* | |
| 1. I was offered something to drink or eat | |
| 1. I had ready access to drinking water | |
| 1. I was informed whether I could eat or drink | |
| 1. It was easy to find the toilets | |
| 1. My bed or trolley was comfortable | |
| 1. My bed did not cause me physical problems such as back pains or sore skin | |
| 1. I was offered additional clothes to go home in | |
| 1. I was offered pillows and blankets | |
| 1. The A&E department was clean and tidy | |
| 1. The temperature in A&E was just about right | |
| 1. Staff did not have to spend time looking for pieces of equipment. | |
| 1. Signs were easy to read | |
| 1. The A&E department was not too noisy | |
| 1. The A&E department was not too bright | |
| 1. I was able to get some sleep if I desired | |
| 1. Candidate items related to *perceptions of the A&E team* |  |
| 1. The whole team displayed kindness towards me |  |
| 1. The A&E team is helpful and acted in a professional way |  |
| 1. The A&E team were respectful and polite |  |
| 1. Members of the team such as house keeping staff and cleaners were helpful |  |
| 1. Members of the team appeared well rested |  |
| 1. Staff wore uniforms and / or badges which made it easy to identify their role. |  |
| 1. I was given the name of a key member of staff when I arrived in the department |  |
| 1. It was clear to me that the A&E team communicated well with each other |  |
| 1. The team worked in a way that was well organised |  |
| 1. My A&E journey was efficient |  |
