# Supplementary material for: Involving patients and caregivers to develop items for a new patient‐reported experience measure for older adults attending the emergency department. Findings from a nominal group technique study
Source: Health Expect. 2023 Jun 30;26(5):2040–9. doi: 10.1111/hex.13811 (PMC10485325; doi:10.1111/hex.13811)
Supplement: Supplementary file 2 — Supporting information. [file HEX-26--s002.docx]

|  |  |  |  |
| --- | --- | --- | --- |
| **Table S2: Final prioritised list of candidate items for inclusion in PREM ED 65+** | | | |
| 1. **Items included via Initial Prioritisation (presented in rank order based on median priority and inter-rater agreement (MADM).** | | | |
|  | **Initial Prioritisation** | | Adjudication |
| Item | **Median**  **Priority** | **MADM** | Favourable Votes, % |
| Staff who were learning were always supervised. | **9** | **0.11** | - |
| The pain relief medicine worked well. | **9** | **0.19** | - |
| I could trust the A&E staff | **9** | **0.3** | - |
| Pain relief medicine was brought to me quickly | **9** | **0.3** | - |
| Staff were thorough and paid attention to the finer details | **9** | **0.33** | - |
| Someone asked me about my views on being revived should my heart stop | **9** | **0.44** | - |
| The A&E team were respectful and polite | **9** | **0.46** | - |
| My disability did not get in the way of my care | **9** | **0.46** | - |
| I felt like staff had reached the right diagnosis | **9** | **0.48** | - |
| Staff undertook checks to make sure my skin was not at risk of damage | **9** | **0.48** | - |
| I did not feel intimidated by the other patients in A&E | **9** | **0.48** | - |
| Staff did not have to spend time looking for equipment | **9** | **0.52** | - |
| Staff made effort to relieve my shortness of breath | **9** | **0.56** | - |
| Staff were quick to respond when I asked for help with the toilet | **9** | **0.56** | - |
| My dignity was always protected. | **9** | **0.59** | - |
| There were enough staff on duty | **9** | **0.59** |  |
| My bed or trolley was comfortable | **9** | **0.59** | - |
| I was given a call bell or other means of summoning help | **9** | **0.59** | - |
| I understood what was being said | **9** | **0.61** | - |
| Staff helped me feel sure that I would be able to cope at home. | **9** | **0.61** |  |
| Staff let me know when to return if things get worse. | **9** | **0.61** | - |
| Staff told me whether I could take my usual medications whilst in A&E | **9** | **0.63** | - |
| Staff were competent | **9** | **0.64** | - |
| Staff explained the risks of tests and procedures | **9** | **0.64** | - |
| Staff let me know in advance when a procedure was likely to be painful or cause discomfort | **9** | **0.64** | - |
| I had confidence in the care I received | **9** | **0.64** | - |
| Staff asked for my consent before they did anything | **9** | **0.66** | - |
| Staff spoke to me as a | **9** | **0.75** | - |
| Staff ensured that I had heard what they had said | **9** | **0.75** | - |
| The A&E team is helpful and acted in a professional way | **9** | **0.78** | - |
| Someone asked me for my views on life support treatment should my condition get worse | **9** | **0.85** | - |
| Staff gave me a discharge letter to give to my GP | **9** | **0.86** | - |
| I had adequate privacy during my A&E stay | **9** | **0.89** | - |
| My A&E journey was efficient | **9** | **0.96** | - |
| Staff were friendly and cheerful. | **9** | **1** | - |
| Staff identified my hidden disability | **9** | **1** | - |
| The whole team displayed kindness towards me | **9** | **1.04** |  |
| Staff were kind | **9** | **1.04** | - |
| Staff met my cultural and language needs. | **8.5** | **1** | - |
| Staff gave me clear discharge instructions | **8** | **0.58** | - |
| Staff let me know why I needed a procedure. | **8** | **0.59** | - |
| Staff explained everything in enough detail. | **8** | **0.68** | - |
| Staff took notice of my long term conditions | **8** | **0.74** | - |
| I was asked how much pain I was in | **8** | **0.78** | - |
| Staff informed me when they were unsure | **8** | **0.79** | - |
| Staff informed me why I was being admitted | **8** | **0.79** | - |
| My relatives or carers did not get in the way of my care. | **8** | **0.81** | - |
| I was able to make my own decisions about my care | **8** | **0.82** | - |
| My pain levels were checked more than once | **8** | **0.85** | - |
| Staff made effort to relieve my other symptoms | **8** | **0.85** | - |
| I was monitored and observed for the right amount of time | **8** | **0.85** | - |
| Staff introduced themselves by name | **8** | **0.86** | - |
| I was helped to the toilet | **8** | **0.93** | - |
| I was told why I needed medicine and about side effects | **8** | **0.96** | - |
| Staff were quick to respond to my problems | **8** | **0.96** | - |
| I received regular updates | **8** | **0.96** | - |
| Staff explained the possible outcomes of tests | **8** | **1** | - |
| Staff cared for my emotional needs. | **8** | **1** |  |
| I felt safe to be discharged | **8** | **1.04** | - |
| Staff were attentive to my needs | **8** | **1.04** | - |
| Signs were easy to read | **8** | **1.04** |  |
| The pain I felt during procedures was about the same, or was less, than I was initially told | **7** | **0.78** | - |
| Staff asked about my ideas, my concerns and  my expectations of care. | **7** | **0.85** | - |
| Staff informed me if something did not go to plan | **7** | **0.86** |  |
| Staff made sure I got exactly what I needed | **7** | **0.89** | - |
| The temperature in A&E was about right | **7** | **0.93** | - |
| Staff understood my worries and concerns | **7** | **0.93** | - |
| Staff reassured me | **7** | **1** |  |
| Staff explained what they were doing to me | **7** | **1** | - |
| I could ask the questions I wanted | **7** | **1.04** |  |
| 1. **Items included via final adjudication (presented in rank order based on proportion of favourable votes (threshold >75%))** | | | |
|  | Initial Prioritisation | | **Adjudication** |
| Item | Median  Priority | MADM | **Favourable Votes, %** |
| I felt like I was safe during my A&E stay | 9 | 1.07 | **100** |
| My bed did not cause me physical problems such as back pains or sore skin | 8 | 1.11 | **100** |
| I felt like I was treated with respect | 8 | 1.11 | **100** |
| I was informed whether I could eat or drink | 8 | 1.33 | **100** |
| The A&E department was clean and tidy | 8 | 1.48 | **100** |
| I had ready access to drinking water | 8 | 1.59 | **100** |
| I was looked after whilst waiting | 7 | 1.26 | **100** |
| I was offered pillows and blankets | 7 | 1.44 | **100** |
| Staff kept me informed about waiting times | 6 | 1.41 | **100** |
| I did not feel lonely during my time in A&E | 9 | 1.11 | **96** |
| Staff explained what I was required to do during a procedure | 9 | 1.30 | **96** |
| Staff checked that I understood what they had said | 8 | 1.11 | **96** |
| Staff involved my relatives or carers as much as I wanted | 7 | 1.32 | **96** |
| It was easy to find the toilets | 7 | 1.51 | **96** |
| I was able to get some sleep if I desired | 5 | 1.56 | **96** |
| Staff spent enough time speaking with me | 7 | 1.21 | **93** |
| I was offered additional clothes to go home in | 7 | 1.40 | **93** |
| Staff let me know what the diagnosis might be. | 7.5 | 1.29 | **89** |
| Staff understood my | 7 | 1.25 | **89** |
| Staff let me know how sick I was | 7 | 1.43 | **89** |
| I felt safe and secure whilst waiting | 7 | 1.64 | **89** |
| I did not feel vulnerable during my A&E stay | 7 | 1.56 | **85** |
| The waiting room was calm, relaxed and pleasant | 5 | 1.19 | **85** |
| Staff checked how I would like to be addressed | 7 | 1.18 | **81** |
| Reception desks were easy to find | 7 | 1.37 | **81** |
| Answers to my questions were clear | 7 | 1.46 | **81** |
| I was given some choice about the type of medicine to take, such as tablets or a drip | 7 | 1.22 | **78** |
| The A&E department was not too bright | 6.5 | 0.9 | **78** |
| I felt like I was a priority | 6 | 1.48 | **78** |
| **Items excluded via final adjudication (presented in rank order based on proportion of favourable votes (threshold <75%))** | | | |
| Item | Median  Priority | MADM | **Favourable Votes, %** |
| I did not feel like I was treated differently because of age | 8 | 1.18 | **74** |
| Information helped to reassure me | 8 | 1.57 | **74** |
| I felt able to make my own choices about my care | 8 | 1.54 | **70** |
| I did not have to wait too long for tests or procedures | 6 | 1.04 | **70** |
| I did not leave A&E feeling frightened or scared about my condition | 6 | 1.44 | **70** |
| Being in A&E was stressful for my relatives or carers | 6 | 1.78 | **70** |
| I was given a say in whether I was admitted or discharged | 3 | 1.93 | **70** |
| Staff explained what is likely to be causing my symptoms | 9 | 1.19 | **67** |
| I was not required to wait in the corridor for a long period | 8 | 1.30 | **67** |
| I could see usually see a clock if I wanted to check the time | 6 | 1.70 | **67** |
| There were enough seats in the waiting room | 7 | 1.19 | **56** |
| The team worked in a way that was well organised | 7 | 2.04 | **56** |
| The A&E department was not too noisy | 6 | 1.26 | **56** |
| I was given an estimate of how long I would have to wait when I was seen at triage. | 5 | 1.93 | **52** |
| The A&E team communicated well with each other | 7 | 1.22 | **51** |
| Leaflets or pictures were used to help me understand | 7 | 1.36 | **51** |
| Staff gave me a leaflet or information sheet to take home. | 6 | 1.46 | **51** |
| There were enough windows and natural light in A&E | 4 | 1.81 | **51** |
| Staff made the right amount of eye contact. | 6 | 0.92 | **44** |
| I was offered something to drink or eat | 3 | 2.11 | **44** |
| I was encouraged to walk around if I wanted to | 4 | 2.14 | **41** |
| Staff told me when I should be well enough to get back to my normal level of mobility. | 6 | 1.61 | **37** |
| I was aware of how busy the rest of the A&E department was, whilst waiting | 6 | 1.78 | **37** |
| The waiting room chairs were comfortable | 5 | 1.89 | **37** |
| There were activities for me to do whilst waiting | 4 | 1.96 | **37** |
| Staff wore uniforms and / or badges which made it easy to identify their role. | 7 | 1.67 | **33** |
| I did not have to repeat myself to many different staff. | 6 | 1.32 | **33** |
| Staff told me when I should be well enough to get back to the things I normally do in life. | 5.5 | 1.89 | **33** |
| I was given the name of a key member of staff when I arrived in the department | 4.5 | 2.11 | **33** |
| Members of the team such as house-keeping staff and cleaners were helpful | 7 | 1.56 | **30** |
| Members of the team appeared well rested | 6 | 1.59 | **30** |
| Staff had a good sense of humour. | 4 | 1.68 | **30** |
| I was informed whether I was likely to be admitted or discharged home | 3 | 1.93 | **30** |
| I was helped to feel in control of my own situation | 6 | 1.07 | **22** |
| Waiting in A&E was not too frustrating | 5 | 2.11 | **22** |
| I was aware of how the urgency of my problem compared to other patients also in A&E. | 4 | 2.11 | **15** |
| Staff recognised if I had a special event such as a birthday | 2 | 1.82 | **11** |
| The department was not too busy or hectic | 5 | 2.15 | **0** |
| I could chat with other patients if I wanted | 1 | 1.11 | **0** |
